# Transfection of *Capsaspora owczarzaki*, a close unicellular relative of animals

**DOI:** 10.1242/dev.162107

**Published:** 2018-05-23

**Authors:** Helena Parra-Acero, Núria Ros-Rocher, Alberto Perez-Posada, Aleksandra Kożyczkowska, Núria Sánchez-Pons, Azusa Nakata, Hiroshi Suga, Sebastián R. Najle, Iñaki Ruiz-Trillo

**Affiliations:** 1 Institut de Biologia Evolutiva (CSIC-Universitat Pompeu Fabra), Passeig Marítim de la Barceloneta 37-49, 08003 Barcelona, Catalonia, Spain; 2 Faculty of Life and Environmental Sciences, Prefectural University of Hiroshima, Nanatsuka 5562, Shobara, Hiroshima 727-0023, Japan; 3 Instituto de Biología Molecular y Celular de Rosario (IBR-CONICET) and Facultad de Ciencias Bioquímicas y Farmacéuticas, Universidad Nacional de Rosario, Ocampo y Esmeralda s/n, Rosario S2000FHQ, Argentina; 4 Departament de Genètica, Microbiologia i Estadística, Universitat de Barcelona, Av. Diagonal, 645, 08028 Barcelona, Catalonia, Spain; 5 ICREA, Passeig Lluís Companys 23, 08010, Barcelona, Catalonia, Spain

**Keywords:** Transfection, *Capsaspora owczarzaki*, Holozoa, Multicellularity, Origin of Metazoa

## Abstract

How animals emerged from their unicellular ancestor remains a major evolutionary question. New genome data from the closest unicellular relatives of animals have provided important insights into the evolution of animal multicellularity. We know that the unicellular ancestor of animals had an unexpectedly complex genetic repertoire, including many genes that are key to animal development and multicellularity. Thus, assessing the function of these genes among unicellular relatives of animals is key to understanding how they were co-opted at the onset of the Metazoa. However, such analyses have been hampered by the lack of genetic tools. Progress has been made in choanoflagellates and teretosporeans, two of the three lineages closely related to animals, whereas no tools are yet available for functional analysis in the third lineage: the filastereans. Importantly, filastereans have a striking repertoire of genes involved in transcriptional regulation and other developmental processes. Here, we describe a reliable transfection method for the filasterean *Capsaspora owczarzaki*. We also provide a set of constructs for visualising subcellular structures in live cells. These tools convert *Capsaspora* into a unique experimentally tractable organism to use to investigate the origin and evolution of animal multicellularity.

## Abstract (precis)

**Summary:** Development of genetic tools in a close unicellular relative of animals, the filasterean *Capsaspora owczarzaki*, will open new opportunities for functional studies to understand the transition to animal multicellularity.

## INTRODUCTION

The transition to animal multicellularity from a single-celled ancestor is one of the most intriguing events in the history of life (King, 2004; Ruiz-Trillo et al., 2007; Rokas, 2008; Knoll, 2011; Richter and King, 2013; Cavalier-Smith, 2017; Sebé-Pedrós et al., 2017). Analysis of the genomes of extant unicellular relatives of animals, hereafter unicellular holozoans, recently showed that the unicellular ancestor of animals was genetically more complex than previously thought (Sebé-Pedrós et al., 2017). Strikingly, genes thought to be animal specific are now known to be present in unicellular holozoans. These include genes involved in cell adhesion, such as those encoding integrins and cadherins, cell-to-cell communication, such as those encoding tyrosine kinases, and transcriptional regulation, such as the developmental transcription factor *Brachyury* (Sebé-Pedrós et al., 2010, 2011; Sebé-Pedrós et al., 2013a; Nichols et al., 2012; Suga et al., 2012). These findings imply that the co-option of ancestral genes into new functions was an important mechanism for the transition to animal multicellularity. However, understanding how these genes were co-opted will only be possible through functional analyses in extant unicellular relatives of animals.

There are three known lineages of unicellular holozoans: choanoflagellates, teretosporeans (ichthyosporeans and corallochytreans) and filastereans (Torruella et al., 2015; Grau-Bové et al., 2017). These three lineages show very different developmental modes, such as the clonal colony formation of choanoflagellates (Fairclough et al., 2013), the coenocytic growth of teretosporeans (Marshall et al., 2008; Suga and Ruiz-Trillo, 2013) and the aggregative behaviour present in filastereans (Sebé-Pedrós et al., 2013b). To develop a comprehensive view of the transition to multicellularity, we need to understand these three different modes of development. So far, a forward genetics approach has been developed in choanoflagellates, leading to the discovery of *rosetteless*, a gene related to colony formation in *Salpingoeca rosetta* (Levin et al., 2014). Efforts are also underway to develop transfection in choanoflagellates. Transfection has already been developed in the ichthyosporean *Creolimax fragrantissima*, where it allowed the description of synchronous nuclear division during coenocytic development (Suga and Ruiz-Trillo, 2013). To date, however, there are still no genetic tools reported in filastereans.

Recent analysis of the genome, transcriptome, proteome and phosphoproteome of the filasterean amoeba *Capsaspora owczarzaki* (Fig. S1), hereafter *Capsaspora*, provided important insights into the origins of animal multicellularity and the nature of their unicellular ancestor (Suga et al., 2013; Sebé-Pedrós et al., 2016a,b). The *Capsaspora* genome encodes an unexpected set of transcription factors known to be involved in animal development that were previously thought to be metazoan specific, such as NFκB, Runx and T-box (Sebé-Pedrós et al., 2011; Suga et al., 2013; de Mendoza et al., 2013). Similar to animals, these transcription factors are differentially regulated at the transcriptional level and are also differentially phosphorylated during the *Capsaspora* life cycle (Sebé-Pedrós et al., 2016a,b). *Capsaspora* also contains the most complete set of proteins linked to cell–extracellular matrix adhesion (the Integrin adhesome) among unicellular holozoans (Sebé-Pedrós et al., 2010; Suga et al., 2013). This highlights *Capsaspora* as the closest relative of animals in which such genes can be studied.

Here, we present the first protocol for transfecting the filasterean *Capsaspora* with plasmid DNA. The protocol is based on the classical calcium phosphate precipitation method (Graham and van der Eb, 1973), which we coupled with a glycerol shock to increase transfection efficiency. We also constructed a set of expression vectors containing an endogenous promoter and fluorescent reporters that allow labelling of multiple subcellular structures *in vivo*. Altogether, this work provides the key step necessary to perform functional assays requiring foreign nucleic acid delivery, including overexpression, RNA interference and genome editing, rendering *Capsaspora* experimentally tractable towards addressing the transition to animal multicellularity.

## RESULTS AND DISCUSSION

### *Capsaspora* transfection using calcium phosphate precipitation

There are several protocols available for the transient transfection of plasmid DNA for eukaryotic cells. For *Capsaspora*, we initially tried electroporation because it has been successfully used to transiently transfect the ichthyosporean *C. fragrantissima* (Suga and Ruiz-Trillo, 2013). However, we obtained no more than 20 positive cells out of thousands of cells. We additionally tried lipid-based transfection (Felgner et al., 1987) and magnetofection (Buerli et al., 2007; Ensenauer et al., 2011), which have been reported to work in eukaryotic cells that are difficult to transfect. Nevertheless, both approaches resulted in few, if any, positive cells. Finally, we tested the classical calcium phosphate precipitation-based transfection method (Graham and van der Eb, 1973), which has been reported to successfully transfect *Dictyostelium discoideum* (Nellen et al., 1984; Gaudet et al., 2007), an amoebozoan without a cell wall. Given that we initially obtained ∼100 times more cells that were positive cells with the calcium phosphate precipitation protocol than with the other methods, we focused on this protocol to further improve its efficiency.

**Fig. 1.**
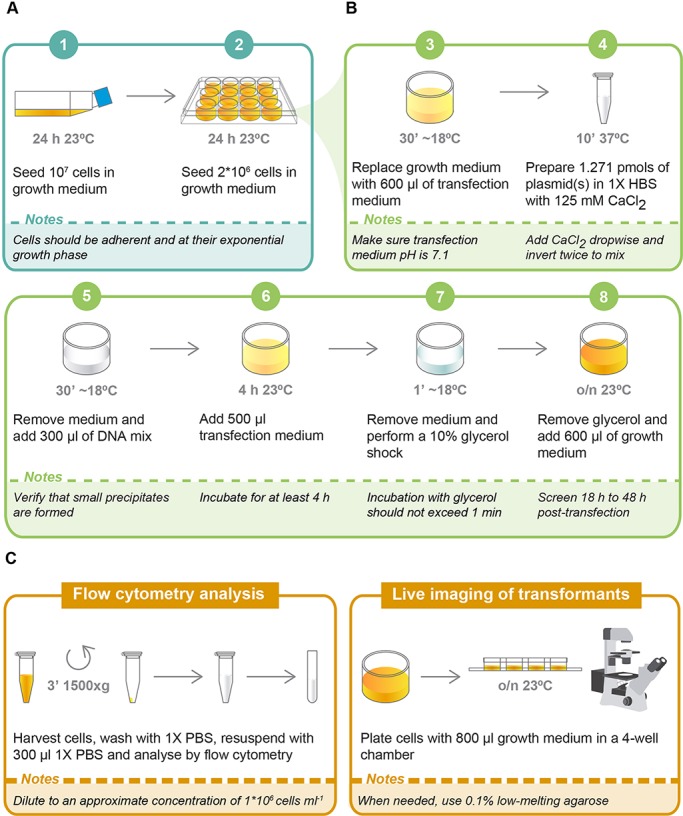
**Protocol for transfection of plasmid DNA in *Capsaspora*.** (A) Preparation of cells (1-2). (B) Calcium phosphate precipitation (3-8). (C) Screening of transformants. o/n, overnight.

As a first step to increase the efficiency of transfection, we investigated which life stage to transfect. Under culture conditions, *Capsaspora* presents three distinct life stages: adherent, cystic and aggregative (Sebé-Pedrós et al., 2013b). We tried using cells in the adherent stage because the culture is at its exponential growth phase at this stage (Fig. 1A) (Sebé-Pedrós et al., 2013b). We observed that transfecting *Capsaspora* adherent cells at 90-95% confluence from a fresh culture resulted in higher transfection efficiency.

**Fig. 2.**
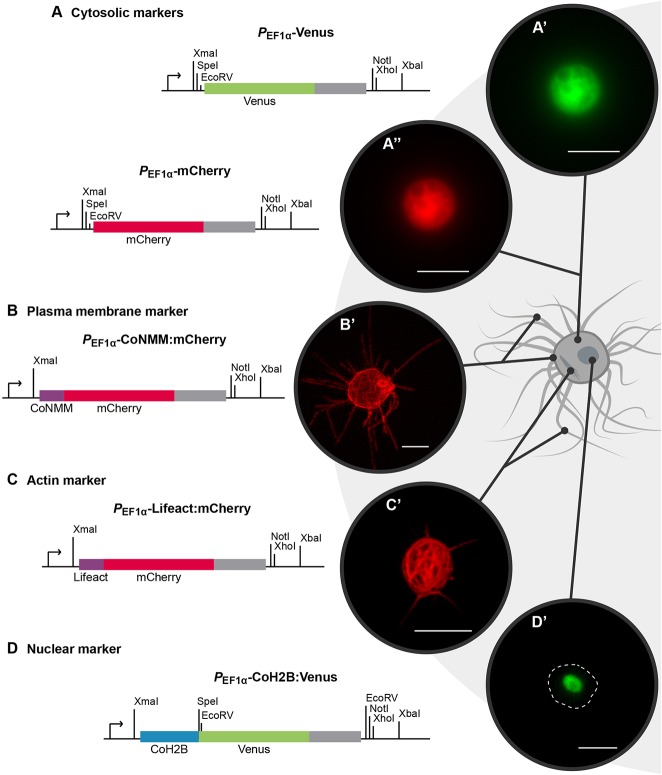
***Capsaspora* expression cassettes and live imaging of transfected cells.** (A) Cytosolic marker cassettes expressing mCherry (A′) or Venus (A″) fluorescent proteins. (B) Plasma membrane marker cassette containing the *Capsaspora Src2* NMM fused to *mCherry* (B′). (C) Actin marker cassette containing *Lifeact* fused to *mCherry* (C′). (D) Nuclear marker cassette containing *Capsaspora* histone *H2B* (*CoH2B*) fused to *Venus* (D′). *EF-1α* promoter (arrows) and terminator (grey boxes) and single-cut restriction enzymes are shown. Cells in (A) and (D) were imaged using wide-field fluorescence microscopy. The cell in B was imaged using a Spinning Disk confocal microscope and the cell in C was imaged using a confocal laser scanning microscope. Dashed line indicates the cell body. Scale bars: 5 μm.

**Fig. 3.**
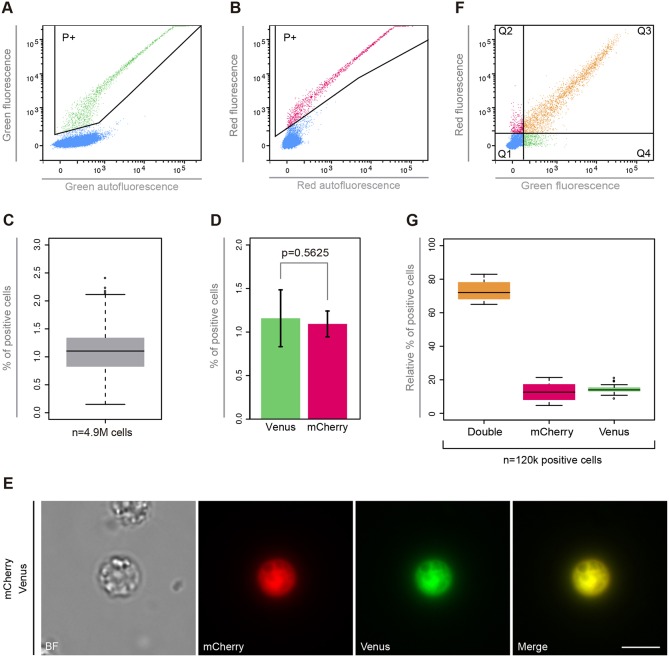
**Transfection efficiency analysis of *Capsaspora*.** (A) Flow cytometry distribution of pONSY-Venus transfected cells. Area selected (P+) represents the Venus-positive population. (B) Flow cytometry distribution of pONSY-mCherry transfected cells. Area selected (P+) represents the mCherry-positive population. (C) Percentage of positive cells in single transfection. The box plot represents the transfection efficiency distribution over seven independent experiments with at least six technical replicates each (*n*=4.9 M cells). (D) Percentage of positive cells from a paired experiment with six technical replicates, transfecting either pONSY-Venus or pONSY-mCherry. Error bars represent s.d. (*P*=0.5625, Wilcoxon Signed Rank Test). (E) Wide-field fluorescence microscopy of a live cell co-transfected with pONSY-Venus and pONSY-mCherry. (F) Flow cytometry distribution of pONSY-Venus and pONSY-mCherry co-transfected cells. Cell population was divided into quartiles: negative cells (Q1), fluorescent cells expressing mCherry only (Q2), co-transfected cells expressing both fluorescent proteins (Q3), and fluorescent cells expressing Venus only (Q4). (G) Relative percentage of positive cells co-transfected with pONSY-Venus and pONSY-mCherry; expressing both fluorescent proteins (double), mCherry only or Venus only, calculated from the total number of positive cells in seven independent experiments with six replicates each (*n*=120,000 cells). Scale bar: 5 µm.

**Fig. 4.**
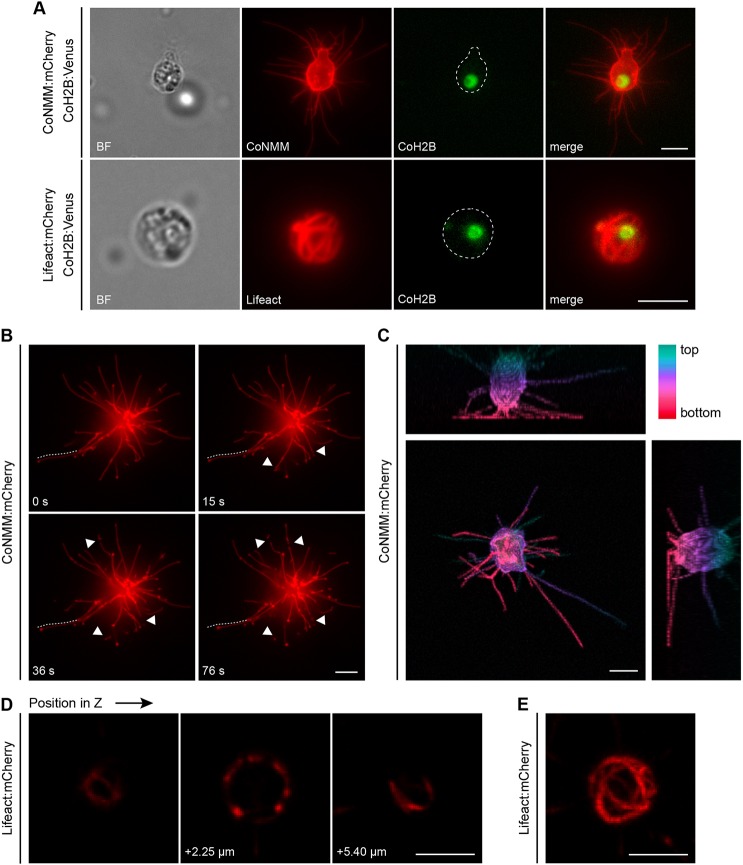
**Live imaging of transfected *Capsaspora* cells.** (A) Wide-field fluorescence microscopy of live cells co-transfected with pONSY-CoNMM:mCherry and pONSY-CoH2B:Venus, and live cells co-transfected with pONSY-Lifeact:mCherry and pONSY-CoH2B:Venus. CoNMM:mCherry labelling is presented as a maximum projection of the cell. Dashed lines indicate cell bodies. (B) Time-points on a *Capsaspora* cell transfected with pONSY-CoNMM:mCherry imaged using wide-field fluorescence microscopy. Filopodia attached to the substrate are in focus. A retracting filopodia can be observed (dotted line), whereas four filopodia are broken (arrowheads). (C) Maximum-intensity projections in each axis of a cell transfected with pONSY-CoNMM:mCherry. Colour scale represents depth through the projection. Imaging was performed using confocal microscopy. (D) z-stack on a *Capsaspora* cell transfected with pONSY-Lifeact:mCherry imaged using confocal microscopy. Actin bundles can be observed shaping the cell in a basket-like structure that is hollow in the middle. (E) Full z-stack maximum intensity projection of cell in (D). Cells in (C-E) were imaged using a Spinning Disk confocal microscope. Scale bars: 5 µm.

Next, we addressed crystal formation during DNA precipitation. We sought the smallest size of crystals possible, because smaller crystals have been associated with higher transfection efficiency (Jordan et al., 1996; Jordan and Wurm, 2004). We achieved this by keeping the same ratio of DNA, calcium and phosphate as previously described for *D. discoideum* (Gaudet et al., 2007), and setting an incubation time of 10 min at 37°C (Fig. 1B-4). Additionally, we used a transfection medium containing minimal growth components but lacking phosphate (Fig. 1B-3,6), to maintain the optimal concentration of calcium phosphate for DNA precipitation. This medium also contains buffering agents at pH 7.1 to avoid pH fluctuations that might affect the solubility of any precipitates.

Finally, to increase the number of transfected cells, we coupled the protocol with a glycerol shock, because the latter has shown to increase the transfection efficiency in other systems (Grosjean et al., 2006; Gaudet et al., 2007; Guo et al., 2017). We performed the shock using 10% glycerol in 1× HBS for 1 min (Fig. 1B-7) and immediately added growth medium to avoid compromising cell viability (Fig. 1B-8, see Supplementary Materials and Methods for further details).

### Analysis of transfected *Capsaspora* cells by flow cytometry

To evaluate DNA incorporation by *Capsaspora*, we constructed two expression vectors containing either Venus (pONSY-Venus) or mCherry (pONSY-mCherry) fluorescent proteins as cytosolic markers (Fig. 2A). These vectors contain the promoter and terminator regions of the endogenous *elongation factor 1-α* (*EF1-α*) gene (CAOG_07807) from *Capsaspora* (see Materials and Methods). We confirmed the successful expression of both fluorescent proteins by fluorescence microscopy (Fig. 2A′-A″) and flow cytometry. We performed an immunofluorescence assay on sorted cells and confirmed that the fluorescent population identified was expressing Venus (Fig. S2).

Next, we analysed the transfection efficiency by quantifying the number of positive cells by flow cytometry (Fig. 3A,B). We performed single transfection experiments using either pONSY-Venus or pONSY-mCherry in seven independent experiments (each experiment performed with a different batch of cells) with at least six technical replicates each. In both cases, the positive populations were defined using a negative control (Fig. S3). *Capsaspora* transfection efficiency was 1.132%±0.529 (mean±s.d.) with a 95% confidence interval of (0.983-1.281%) from a total of 4.9 million cells (Fig. 3C). In these experiments, individual transfection efficiencies ranged from 0.347%±0.193 to 2.083%±0.248 (Table S1). Importantly, these transfection efficiencies are sufficient to screen for positive cells and perform further manipulations, because they correspond to thousands of positive cells per well. Additionally, we compared transfection rates between *Capsaspora* cells transfected with either pONSY-Venus or pONSY-mCherry (Fig. 3D, experiments 7a and 7b, respectively, in Table S1), but no significant difference was observed (*P*=0.5625, Wilcoxon Signed Rank Test).

Given that transfection is transient, it is of interest to know how long the expression of the reporter gene persists for. Thus, we performed three independent experiments transfecting pONSY-Venus. We analysed the percentage of positive cells by flow cytometry every 24 h for 10 days (Fig. S4 and Table S2). We observed an exponential decrease in the number of positive cells. Although there was a significant reduction in the number of positive cells after 48 h (∼39% of the total of positive cells), positive cells (∼3%) could still be detected by Day 10. At this point, most of the cells are expected to be in the cystic stage (Sebé-Pedrós et al., 2013b), indicating that transient expression of a gene of interest can be analysed during each of the life stages of *Capsaspora*.

We also tested whether the two different constructs could express a protein simultaneously by co-transfecting both pONSY-mCherry and pONSY-Venus at equimolar concentrations in seven independent experiments (Fig. 3E). The red (Q2) and green (Q4) positive populations were defined using their corresponding negative controls (Fig. S5). The mean relative percentage of cells showing red and green fluorescence simultaneously (Q3 in Fig. 3F) from the total number of positive cells (sum of Q2, Q3 and Q4 in Fig. 3F) was 72.909%±5.468, ranging from ∼65% to ∼83% (Fig. 3G, Table S3). Thus, it is possible to co-transfect two different vectors with a high probability of incorporating both of them in the same cell. This result is similar to those observed in other unicellular eukaryotes [40-80% in *Volvox carteri* (Schiedlmeier et al., 1994), 84% in *Pandorina morum* (Lerche and Hallmann, 2014) and 50-100% in *Eudorina elegans* (Lerche and Hallmann, 2013)]. Co-transfection is a useful strategy when more than one cassette is needed, such as when labelling two different cellular structures simultaneously, delivering resistance cassettes against an antibiotic with a reporter gene, or delivering different elements required for CRISPR/Cas9 assays.

### Live imaging of *Capsaspora* by labelling endogenous proteins

To understand the biological role of certain genes in *Capsaspora*, it is important to subcellularly localise the protein of interest in the cell. Thus, as a means of labelling the cellular structures in *Capsaspora*, we designed three additional vectors that allowed live imaging of the plasma membrane, the actin cytoskeleton and the nucleus*.*

To label the plasma membrane, we built a construct expressing an endogenous membrane-binding motif fused to mCherry (pONSY-CoNMM:mCherry, Fig. 2B). We used the *N*-myristoylation motif (NMM), a well-known membrane-binding motif, present in the *Src* tyrosine kinases (Sigal et al., 1994). *Capsaspora* has two homologs of *Src*, *CoSrc1* (CAOG_02182) and *CoSrc2* (CAOG_06360), the localisation of which has been reported in filopodia (Schultheiss et al., 2012). We used *CoSrc2* NMM to create the CoNMM:mCherry fusion, which successfully localised at the plasma membrane, including filopodia (Fig. 2B′). To label the actin cytoskeleton in *Capsaspora*, we built a construct containing Lifeact fused to mCherry (pONSY-Lifeact:mCherry, Fig. 2C). Lifeact is a 17-amino acid peptide from the N-terminal region of yeast Abp140 (Riedl et al., 2008) that works as a marker of filamentous actin. The Lifeact:mCherry fusion successfully labelled the actin cytoskeleton (Fig. 2C′). This construct also labels actin in filopodia (Fig. S7), although the signal is much weaker than that observed with the membrane marker. Finally, to label the nucleus, we built a construct containing the coding sequence of *Capsaspora* histone *H2B* (CAOG_01818) fused to Venus (pONSY-CoH2B:Venus, Fig. 2D). We confirmed nuclear localisation by staining transfected cells with DAPI (Fig. 2D′, Fig. S6).

To better understand the subcellular structures of *Capsaspora* cells, we combined the nuclear, plasma membrane and actin markers. We co-transfected pONSY-CoH2B:Venus with either pONSY-CoNMM:mCherry or pONSY-Lifeact:mCherry (Fig. 4A). Furthermore, we performed live imaging in cells transfected with either the membrane marker or the actin marker. The use of the membrane marker allowed us to observe the dynamic behaviour of filopodia on the substrate with unprecedented detail. We observed the retraction of filopodia, filopodia breakage and foci of membrane accumulation (Fig. 4B and Movie 1). In particular, we observed that filopodia are distributed around the cell body. More importantly, the projections constructed from the z-stack clearly demonstrated that the *Capsaspora* cell body is not in direct contact with the substrate, with the numerous filopodia instead holding the cell up (Fig. 4C). Moreover, we tracked a cell transfected with the actin cytoskeleton marker and observed the organisation of actin bundles around the cell body (Fig. 4D,E and Movie 2).

The accumulated knowledge on its well-annotated genome, transcriptome, proteome and phosphoproteome and histone modifications, and its key phylogenetic position as a close unicellular relative to animals render *Capsaspora* as a powerful system to understand the emergence of multicellular animals. The reliable transfection protocol for *Capsaspora* presented here will allow us to study the function of genes that were key to the evolution of multicellularity, opening new avenues of functional research to better understand the transition to animal multicellularity.

## MATERIALS AND METHODS

### Cell strain and growth conditions

*C.* *owczarzaki* cell cultures (strain ATCC^®^30864) were grown axenically in 25 cm^2^ culture flasks (Falcon^®^ VWR, #734-0044) with 5 ml ATCC medium 1034 (modified PYNFH medium), hereafter growth medium, in a 23°C incubator (see supplementary Materials and Methods).

### Construction of *Capsaspora* expression vectors

DNA from *Capsaspora* cells was extracted as in Suga et al. (2013). RNA was extracted using a Trizol reagent (Invitrogen/Thermo Fisher Scientific, #15596026). cDNA was obtained by RT-PCR using SuperScript^®^ III Reverse Transcriptase (Invitrogen, #18080044) following the manufacturer's instructions.

*Capsaspora* expression vectors, named pONSY, bear the promoter and terminator regions from the endogenous *EF-1α* gene (CAOG_07807). To build the pONSY-Venus vector (5.849 kb), the *EF-1α* promoter (906 bp upstream from methionine) and terminator (320 bp downstream from the stop codon) were amplified from gDNA with primers 1 and 2 and primers 4 and 5, respectively (Table S4). *Venus* was amplified from a plasmid available in H.P.-A.'s lab using primers 7 and 8, which contain overlap regions with the promoter and terminator regions, respectively. The three amplicons were fused together by overlapping PCR using primers 1 and 5. The resulting *P*_EF1α_-Venus-terminator cassette was digested using the *Kpn*I restriction enzyme and cloned into the pCR2.1 vector (Life Technologies, #K203001) linearised at the *Kpn*I restriction site.

To build pONSY-mCherry (5.828 kb), we followed the same strategy as described above for the promoter, and used primers 4 and 6 to amplify the terminator region. This modification was introduced to eliminate an extra *EcoR*V site that affects further cloning. *mCherry* was amplified from a plasmid available in H.P.-A.'s lab using primers 9 and 10, which contain overlap regions with both the promoter and terminator regions. The three amplicons were fused together by overlapping PCR using primers 1 and 6. The resulting *P*_EF1α_-mCherry-terminator cassette was digested with *Kpn*I and *Ksp*I enzymes and cloned into the respective restriction sites of the pCR2.1 vector.

A pONSY (empty) vector (5.127 kb) was created by releasing a mCherry-terminator fragment from pONSY-mCherry using *Spe*I and *Ksp*I restriction enzymes and inserting the terminator in this backbone by Gibson Assembly^®^ (New England Biolabs, E2611L) using primers 7 and 8.

pONSY-CoH2B:Venus (6.230 kb) was created by fusing *Capsaspora* histone *H2B* (CAOG_01818) to Venus. *CoH2B* was PCR amplified from cDNA using primers 13 and 14 and cloned into the pONSY-Venus multicloning site using *Sma*I and *Spe*I restriction enzymes.

pONSY-CoNMM:mCherry (5.904 kb) was created by fusing an NMM to mCherry. NMM was predicted in the *Capsaspora* Src homolog CoSrc2 (CAOG_06360) using ‘NMT - The MYR Predictor’ online software (http://mendel.imp.ac.at/myristate/SUPLpredictor.htm), which is based on an in-depth study of *N*-myristoyltransferase substrate proteins (Maurer-Stroh et al., 2002). The NMM predicted sequence GCSNSKPHDPSDFKVSP plus seven extra amino acids (SGVASNS) and an *mCherry* overlap region were included in primer 11. Primers 11 and 12 were used to build a CoNMM-mCherry cassette by PCR using the pONSY-mCherry vector as a template. This cassette was then cloned into pONSY (empty) using *Xma*I and *Eco*RV restriction enzymes.

pONSY-Lifeact:mCherry (5.882 kb) was created by fusing the Lifeact peptide MGVADLIKKFESISKEE(GDPP) (linker in parentheses) to mCherry using primers 15 and 16. The codons were optimised according to their usages in *C. owczarzaki* and *C. fragrantissima*. The Lifeact DNA fragment was first cloned into a pTAC-2 vector (BioDynamics Laboratory) by TA cloning, and the *Xma*I- and *Xba*I-excised fragment was cloned into the pONSY:mCherry vector.

All plasmids DNA were obtained using the plasmid GenElute™ Plasmid Midiprep Kit (Sigma, #NA0200-UKT), lyophilised and resuspended at an approximate concentration of 1 µg/μl in distilled water.

### Transfection of *Capsaspora owczarzaki*

*Capsaspora* cells were transfected using a calcium-phosphate DNA precipitation protocol coupled with a glycerol shock. At Day 0, 2×10^6^ cells were seeded in a 12-well plate (Nunc/DDBioLab, #55428) containing growth medium and grown at 23°C overnight.

At Day 1, growth medium was replaced by transfection medium (see Supplementary Materials and Methods), and incubated for 30 min at room temperature (∼18°C). During incubation, the DNA mix was prepared with 1.271 pmol of plasmid DNA for single transfection experiments or 0.636 pmol of each plasmid DNA for co-transfection experiments in 1× HBS Buffer. CaCl_2_ was added dropwise to a final concentration of 125 mM. The DNA mix was inverted immediately twice and incubated for 10 min at 37°C. After incubation, the transfection medium was removed gradually and the DNA mix was added dropwise to the centre of each well. The cell:DNA mixes were incubated for 30 min at room temperature, after which the transfection medium was added to each well. Cells were incubated for 4 h at 23°C. After incubation, medium was removed and an osmotic shock was performed using 10% glycerol in 1× HBS buffer, for 1 min at room temperature. After the osmotic shock, the glycerol solution was replaced by growth medium and cells were incubated at 23°C overnight. Screening of positive cells was performed 18 h post transfection. More details about the transfection protocol and preparation of transfection reagents are listed in the Supplementary Materials and Methods.

### Flow cytometry and FACS

Transfection efficiency was analysed 18 h post transfection. pONSY (empty) transfected cells, mock-transfected cells and nontransfected cells were used as controls for all transfection experiments to discriminate autofluorescence and to distinguish the positive population. For co-transfection experiments, pONSY-mCherry and pONSY-Venus single-transfected controls were used to correctly identify double-positive cells.

Cells were scraped and harvested by centrifugation at 1500 ×***g*** for 3 min at 18°C, washed once with 500 µl 1× PBS (Sigma, #P5368-10 PAK) and diluted to a final concentration of 1×10^6^ cells ml^−1^ in a minimum volume of 300 µl 1× PBS. Samples were analysed by flow cytometry using a BD LSRFortessa analyser (Becton Dickinson).

To evaluate plasmid persistence over time, transfected cells from 12 wells per experiment were pooled to homogenise the sample, were then split again into 12 new wells and grown for 10 days. Samples were scraped and harvested by centrifugation at 1500 ×***g*** for 3 min at 18°C. Samples were fixed with 4% formaldehyde (Sigma-Aldrich, #F8775-4X25ML) in 1× PBS for 10 min at 18°C and washed once with 500 µl 1× PBS (Sigma, #P5368-10 PAK). Finally, cells were re-suspended in 400 µl 1× PBS and kept at 4°C until analysed.

SSC-A and FSC-A parameters were used to detect populations of cells (P1). Single cells were gated by FSC-H and FSC-A (P2). Around 100,000 events were recorded from P2, whenever possible. Venus-positive cells (P+ or Q1) were detected using a 488 nm laser with a 530/28 bandpass filter (green fluorescence) and differentiated from autofluorescent cells with a 670/50 bandpass filter.

mCherry-positive cells (P+ or Q4) were detected using a 561 nm laser with a 610/20 bandpass filter (red fluorescence) and differentiated from autofluorescent cells with a 780/60 bandpass filter. Around 2000 events in the population expressing both Venus and mCherry (Q2) were recorded.

For immunofluorescence validation, pONSY-Venus transfected cells were harvested as before and diluted to a concentration of 1×10^7^ cells ml^−1^ in a minimum volume of 500 µl 1× PBS. Cells from nine replicates were pooled. Then, 40,000 Venus-positive cells (P+) and 1 million Venus-negative cells (P−) were sorted using a BD FACSAria II SORP flow cytometer cell sorter (Becton Dickinson) equipped with a 100 µm nozzle. The cell population (P2) was gated as before. P+ and P− were detected using a 488 nm laser with a 525/50 bandpass filter (green fluorescence) and differentiated from autofluorescent cells with a 605/40 bandpass filter. Flow cytometry data were visualised and analysed using FlowJo software (FlowJo LLC, version 9.9.3).

### Immunostaining

Sorted cells were collected in 200 µl of 1× PBS and seeded in a Nunc glass-bottom dish (Thermo Fisher Scientific, #150680) previously treated with 200 µl of 20 µg ml^−1^ fibronectin (Sigma-Aldrich, #F1141-2MG) overnight at 4°C. Cells were incubated for 3 h at 23°C, then 1× PBS was substituted with 200 µl growth medium and grown overnight at 23°C.

Cells were fixed for 5 min at room temperature with 4% formaldehyde in 1× PBS and washed once with 200 µl 1× PBS. Cells were then blocked for 30 min at room temperature in blocking solution [1% bovine serum albumin (Sigma-Aldrich, #A3294-10G), 0.1% Triton-X100 (Sigma-Aldrich, X100) in 1× PBS] and incubated for 1.5 h at room temperature with 1:100 anti-green fluorescent protein (GFP) primary antibody (Abcam, ab5450, Lot GR277059-1) in blocking solution [Venus is an improved version of GFP (Nagai et al., 2002)]. Cells were washed twice for 10 min in blocking solution and incubated for 1.5 h in the dark at room temperature with 1:1000 Alexa Fluor 568 goat anti-rat IgG (Life Technologies, A11077, Lot 1512105) in the blocking solution. After three washes of 10 min in 1× PBS, the preparation was overlaid with fluorescence mounting media (DAKO/Agilent Technologies, #S3023), covered with a coverslip and sealed with nail polish.

### Imaging of transfected cells

Immunostained samples were imaged using a Leica TCS SP5 II inverted confocal microscope with a 63× immersion oil objective. Acquisition settings were adjusted using Venus-positive cells without primary antibody and Venus-negative cells.

For live imaging, all samples were plated in a µ-Slide 4-well glass-bottom dish (Ibidi, #80427) and grown overnight at 23°C. In the case of pONSY-H2B:Venus transfected cell samples, plated cells were washed once with 200 µl 1× PBS, fixed for 5 min at room temperature with 4% formaldehyde in 1× PBS and washed again as before. Cells were covered using Vectashield with DAPI (Vector, #H-1200). For live imaging of the cytoskeleton, cells were plated in a µ-Slide 4-well Ph+ glass-bottom dish (Ibidi, #80447) in 800 µl of growth medium containing 0.1% low-melting agarose (Sudelab #8085). Wide-field microscopy was performed using a Zeiss Axio Observer Z.1 epifluorescence inverted microscope equipped with LED illumination and a Axiocam 503 mono camera.

Time-lapse videos were recorded using the same microscope. For Movie 1, acquisition was performed at 1 frame/s (fps) and video export was performed at 10 fps. For Movie 2, images were taken every 10 min and video export was performed at 2 fps. A maximum intensity projection was used of two slices from a *z*-stack.

When indicated, membrane and cytoskeleton labelling were additionally imaged using confocal microscopy with an Andor Revolution XD Spinning Disk microscope equipped with an Andor Ixon 897E Dual Mode EM-CCD camera. These images were deconvolved using The Huygens System 17.10-64 Multi-Processing edition software.

Confocal microscopy was performed with a 63× immersion oil objective using either a confocal laser scanning Leica TCS SP5 II microscope or an Andor Revolution XD Spinning Disk microscope equipped with an Andor Ixon 897E Dual Mode EM-CCD camera. These images were deconvolved using The Huygens System 17.10-64 Multi-Processing edition software.

All images were edited using Fiji Imaging Software version 2.0.0-rc-44/1.50e (Schindelin et al., 2012).

### Statistical analysis

Results are shown as mean±standard deviation (s.d.) per experiment. The 95% confidence intervals were calculated using the Student's *t*-test. The significance of differences in the percentage of positive cells from single-transfection experiments were tested using the non-parametric Wilcoxon Signed Rank Test for paired samples. All statistical analyses were performed using the R Stats Package version 3.3.1 (R Core Team, 2016).

## Supplementary Material

Supplementary information
