# Supplementary material for: Transfection of Capsaspora owczarzaki, a close unicellular relative of animals
Source: Development. 2018 May 23;145(10):dev162107. doi: 10.1242/dev.162107 (PMC6001378; doi:10.1242/dev.162107)
Supplement: Supplementary information [file develop-145-162107-s1.pdf]

Supplementary Materials and Methods

Transfection of *Capsaspora* using Calcium-phosphate precipitation

Adherent stage cells at the exponential growth phase were obtained after a two-day culture passage as follows. Two days before transfection, $1x10^7$ cells were seeded in a 25 cm^2 culture flask containing 5 mL growth medium and grown overnight at 23ºC (Fig. 1A-1). Hereafter, all amounts are indicated per well.

At day 0, $2x10^6$ cells were seeded from this previous confluent culture to attain 90-95% cell confluence at the time of transfection. Cells were seeded in a 12-well plate (Nunc/DDBioLab #55428) containing 600 µL growth medium and grown overnight at 23ºC (Fig. 1A-2). Cell concentration was determined using a Neubauer Chamber Hemocytometer (DDBiolab #900505).

⚠ ***CRITICAL STEP: Adherent stage cells in confluency.*** *Cultures should be fresh to maximize transfection efficiency. Ideally, they should be maintained weekly, and used for transfection at their exponential growth phase. Do not let cultures reach higher cell densities (< $5x10^7$ cells mL^{-1}).*

At day 1, growth medium was replaced by 600 µL of transfection medium (see Reagent preparation), and incubated for 30 min at room temperature (~18ºC) (Fig. 1B-3). During incubation, 1.271 pmols of plasmid DNA for single transfection experiments or 0.636 pmols of each plasmid DNA for co-transfection experiments were diluted in sterile distilled water up to 120 µL plus an additional volume of 150 µL of 2X HBS Buffer. Next, 30 µL of 1.25 M $CaCl_2$ were added dropwise while flickering the tube carefully, reaching a final DNA mix volume of 300 µL. DNA mix was inverted immediately two times to ensure proper mixing of reagents and incubated 10 min at 37ºC (Fig. 1B-4). After incubation, transfection medium was removed and the DNA mix was added dropwise in the centre of the wells. Cells:DNA mix were incubated for 30 min at 18ºC (Fig. 1B-5).

⚠ ***CRITICAL STEP: DNA-Calcium-phosphate precipitates formation.*** *Check the cultures periodically under the microscope to check crystal size. Big cloudy precipitates may compromise transfection efficiency. Instead, verify that small grains of refractant material are spread homogeneously in the plate.*

After this period, an additional volume of 500 µL of transfection medium was added and cells were incubated for a minimum of 4 h at 23ºC (Fig. 1B-6).

⚠ ***NOTE: Transfection medium incubation.*** An incubation of less than 4 h yields lower transfection efficiency. This incubation time can be extended to 6 h.

Development • Supplementary information

After incubation, the medium was removed and an osmotic shock using 110 µL 10% (v/v) glycerol in 1X HBS Buffer was performed, pouring the solution dropwise all over the well for one min at ~18ºC (Fig. 1B-7).

⍍ ***CRITICAL STEP: Glycerol shock.*** *Incubation with glycerol at this concentration should not exceed 1 min, counting from the first droplet, to avoid excessive cell death.*

After the osmotic shock, glycerol solution was removed and cells were grown at 23ºC overnight with 700 µL of growth medium (Fig. 1B-8). Screening of positive cells was performed 18 h post-transfection using fluorescence microscopy and flow cytometry analysis (Fig. 1C).

⍍ ***NOTE: Controls.*** pONSY (empty) transfected cells, mock-transfected cells and non-transfected cells were used as controls.

Development • Supplementary information

Transfection Reagents preparation

Growth medium (for 1 L): 10 g Peptone (BD, #211677), 10 g Yeast Extract (BD, #212750), 1 g Yeast nucleic acid (Ribonucleic Acid, Type VI from Torula Yeast) (Sigma, #R-6625), 15 mg Folic acid (Sigma, #F8758) in 880 mL distilled water. Autoclave for 15 min at 121ºC. Cool down and aseptically add 0.4 mL of Hemin stock solution* (Sigma, #H9039), 20 mL Buffer solution** and 100 mL of heat-inactivated Fetal Bovine Serum (Sigma, #F9665-100ml). Filter-sterilise through 0.22 µm and store at 4ºC.

***Hemin stock solution (for 200 mL):** 400 mg NaOH in 200 mL dH_2O. Add 500 mg of Hemin and autoclave 20 min at 121ºC. Store at 4ºC protected from the light.

****Buffer solution (for 1 L):** 18.1 g KH_2PO_4 (Sigma, #P5655), 25 g Na_2HPO_4 (Sigma, #S5136) in 1 L distilled water. Adjust final pH to 6.5 with HCl 37% and filter-sterilise through 0.22 µm. Store at 4ºC.

Transfection medium (for 1 L): 10 g Peptone, 15 mg Folic Acid in 990 mL distilled water. Autoclave for 20 min at 121ºC. Aseptically add 10 mL HEPES 1 M (Sigma, #H4034) to a final concentration of 10 mM and 2.1 g Bis-Tris methane (Sigma, #B9754) final concentration 0.21% w/w. adjust pH to 7.1 with NaOH, filter-sterilise through 0.22 µm and store at 4ºC.

2X HBS (for 250 mL): Dissolve 4 g NaCl (Sigma, #S3014), 0.18 g KCl (Sigma, #P9541), 0.05 g Na_2HPO_4 (Sigma, #S5136), 2.5 g HEPES and 0.5 g D-glucose (Sigma, #G8270) in autoclaved distilled water. Adjust pH to 7.1 with NaOH. Filter-sterilise through 0.22 µm, flash-freeze with liquid Nitrogen and store at -80ºC.

1.25M $CaCl_2$ (for 10 mL): 1.84 g $CaCl_2$ (Sigma, #C1016) in 10 mL autoclaved distilled water. Filter-sterilise through 0.22 µm, flash-freeze with liquid Nitrogen and store at -80ºC.

10% glycerol (for 4 mL): 0.8 mL of filter-sterilised 50% (v/v) glycerol (Sigma, #G7757) in 1.2 mL autoclaved distilled water and 2 mL 2X HBS. Filter-sterilise through 0.22 µm, flash-freeze with liquid Nitrogen and store at -80ºC.

Supplementary Figures

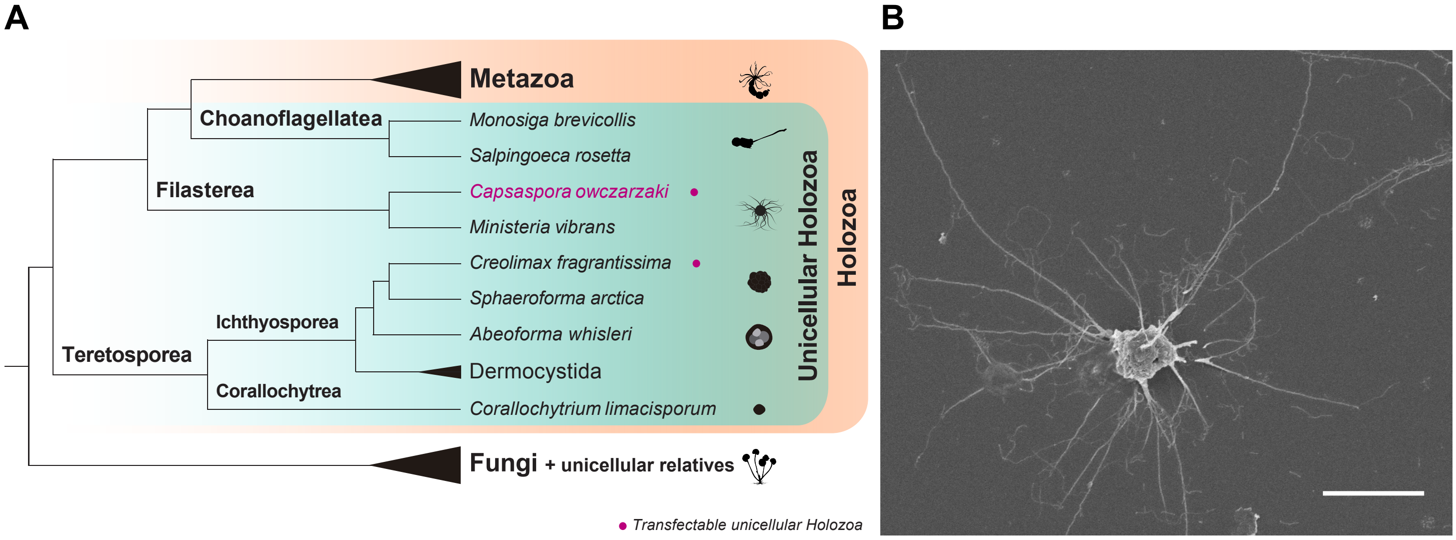

Fig. S1. Transfectable unicellular Holozoa and *Capsaspora owczarzaki*. (A) Metazoa and their unicellular relatives; Choanoflagellatea, Filasterea and Teretosporea, comprise the Holozoa clade. Transfectable unicellular Holozoa to date are *C. fragrantissima* and *C. owczarzaki.* (B) SEM image of a *Capsaspora* cell*.* Scale bar represents 5 µm.

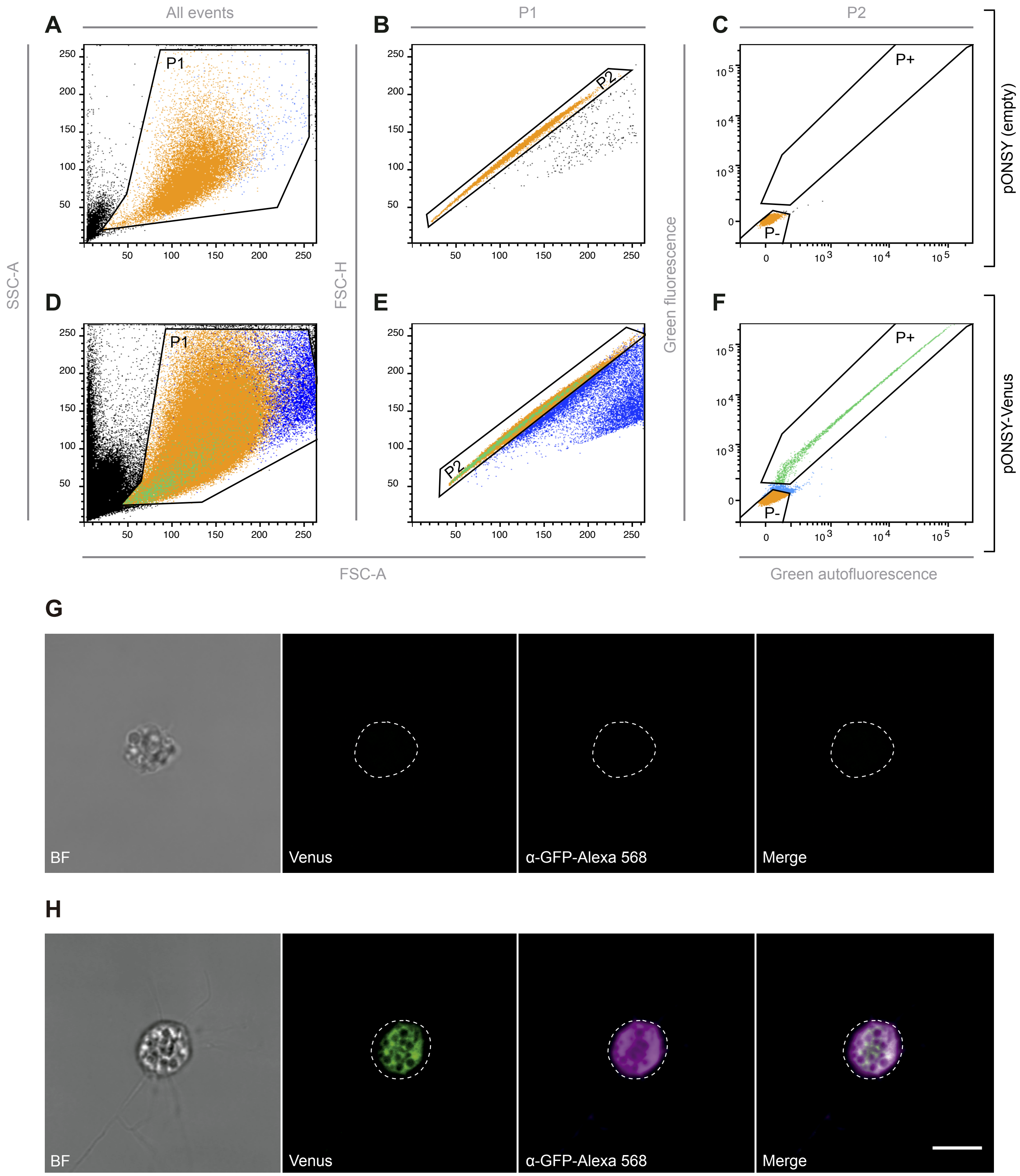

Fig. S2. FACS of *Capsaspora* transfected cells and immunofluorescence validation. (A-C) Cells transfected with pONSY (empty) as control to gate positive and negative populations. (D-F) Cells transfected with pONSY-Venus. Areas selected in (A) and (D) define total population of cells (P1). Areas selected in (B) and (E) define single cells (P2). Areas in (C) and (F) define sorted Venus positive cells (P+) and sorted Venus negative cells (P-), respectively. (G-H) Immunofluorescence validation of Venus expression of P- (G) and P+ (H) sorted populations from (F) using an anti-GFP antibody. Dashed line indicates cell body. Scale bar represents 5 μm.

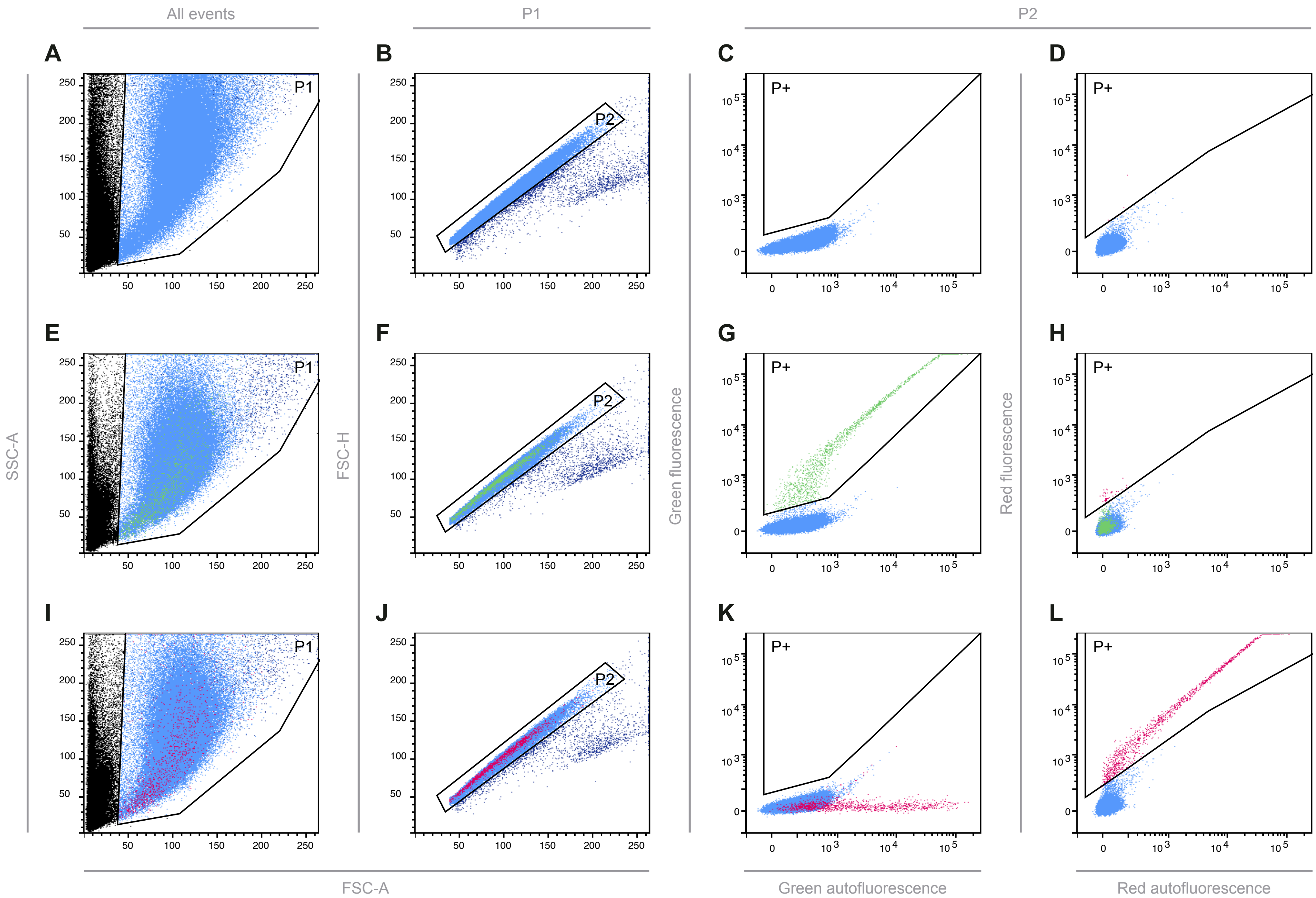

Fig. S3. Flow cytometry analysis of *Capsaspora* transfected cells. (A-D) Cells transfected with pONSY (empty) as control to gate positive and negative populations. (E-H) Cells transfected with pONSY-Venus. (I-L) Cells transfected with pONSY-mCherry. Areas selected in A, E and I define total population of cells (P1). Areas selected in B, F and J define single cells (P2). P+ in C, G and K defines positive cells in the green channel (Venus). P+ in D, H and L defines positive cells in the red channel (mCherry). Figure associated to Fig. 3A-B.

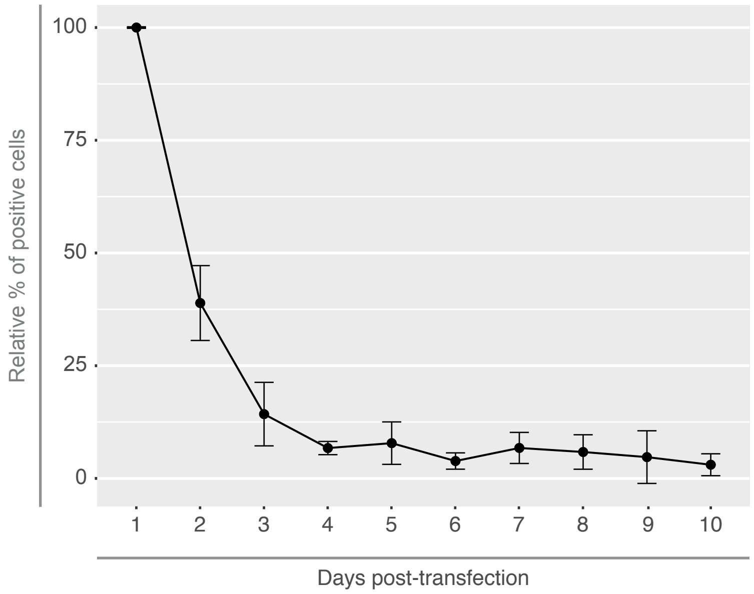

Fig. S4: Persistance of positive cells along 10 days after transfection. Percentage of positive cells transfected with pONSY-Venus, measured every 24h by flow cytometry (number of positive cells at day 1 was considered as 100%). Error bars represent s.d. Figure associated to Table S2.

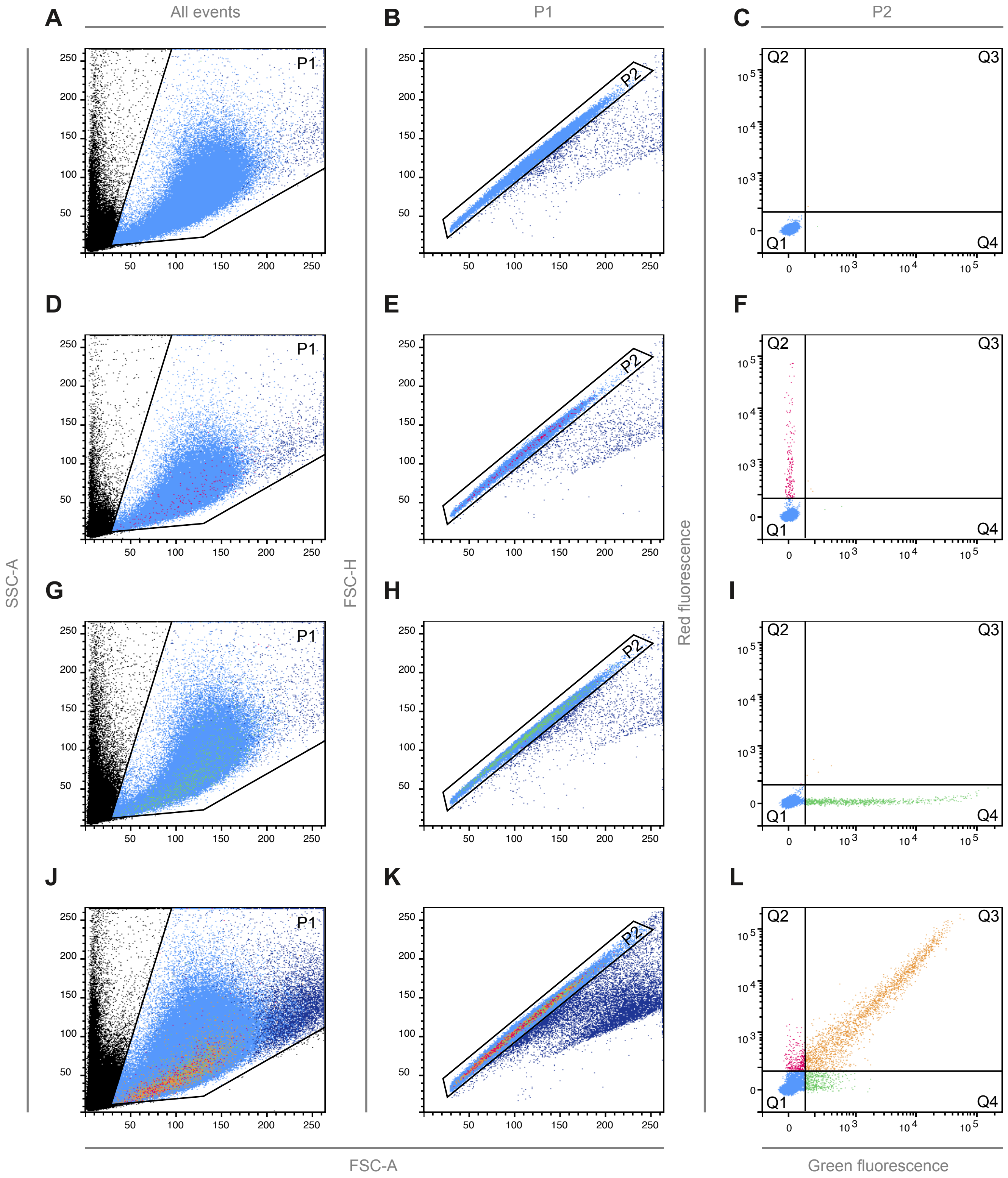

Fig. S5: *Capsaspora* co-transfected with both pONSY-mCherry and pONSY-Venus. (A-C) Cells transfected with pONSY (empty) as control. (D-F) Cells transfected with pONSY-mCherry only. (G-H) Cells transfected with pONSY-Venus only. (J-L) Cells co-transfected with both pONSY-mCherry and pONSY-Venus. Areas selected in panels A,D,G and J define total population of cells (P1). Areas selected in B, E, H, and K define single cells (P2). Quartiles define negative cells (Q1), red fluorescent cells expressing mCherry only (Q2), cells expressing both fluorescent proteins (Q3) and green fluorescent cells expressing Venus only (Q4). Figure associated to Fig. 3F.

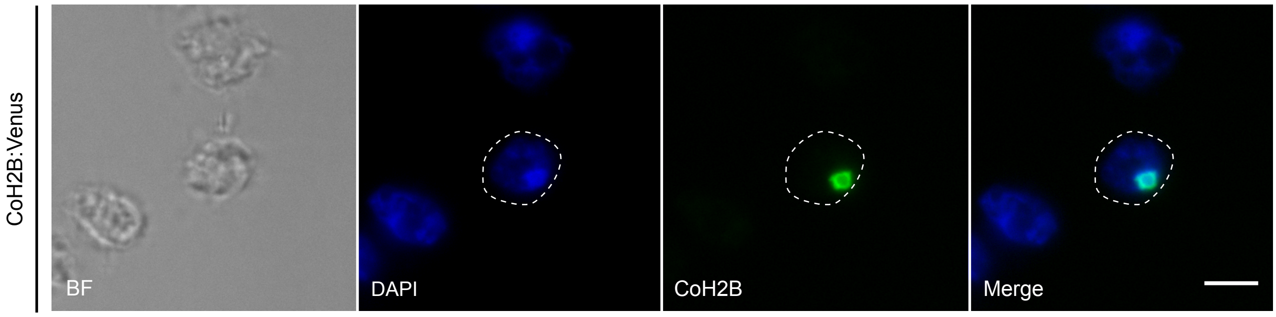

Fig. S6. Localisation of nuclear marker in *Capsaspora* transfected cells. Transfected cells with pONSY-CoH2B:Venus stained with DAPI. Dashed line indicates cell body. Scale bar represents 5 µm.

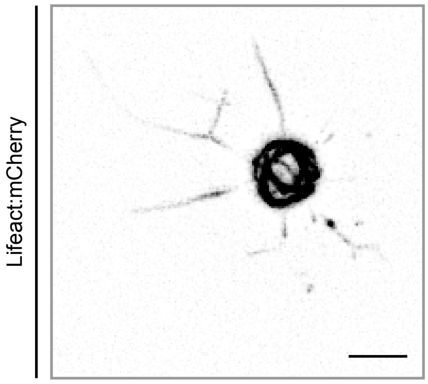

Fig. S7. Labelling the actin cytoskeleton and filopodia in *Capsaspora*. Transfected cell with pONSY-Lifeact:mCherry from Fig. 2C'. Image saturated and inverted to improve visualization of filopodia. Scale bar represents 5 μm.

Supplementary movies

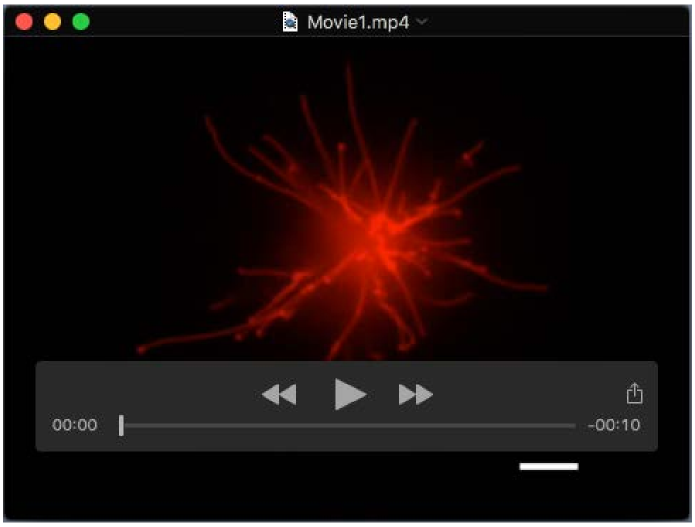

Movie 1. *Capsaspora* filopodia dynamics *in vivo*. Time-lapse of a cell transfected with pONSY:CoNMM-mCherry. Images were taken every second during 100 seconds. Scale bar represents 5 µm.

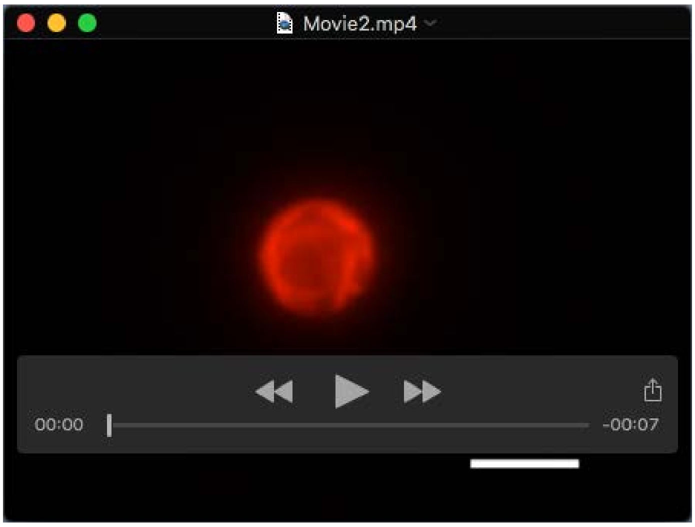

Movie 2. *Capsaspora* actin cytoskeleton *in vivo*. Time-lapse of a cell transfected with pONSY:Lifeact-mCherry. Images were taken every 10 minutes during 130 minutes. Scale bar represents 5 µm.

Supplementary Tables

Tables S1-S4

Table S1. Flow Cytometry analysis of *Capsaspora* cells transfected with a single vector. Flow cytometry analysis of *Capsaspora* cells transfected with pONSY-Venus (1-7a) or pONSY-mCherry (7b) expression vectors. Results from 7 independent experiments with at least 6 replicates each (n=51) are shown. Transfection efficiency is calculated as the ratio of total number of positive cells (P+) from total number of cells (P2) and represented as mean±s.d per experiment. Table associated to Fig. 3A-D and Fig. S3.

Experiment		Number of cells		Transfection efficiency	
**Number**	**Sample**	**Total (P2)**	**Positive (P+)**	**(P+/P2)%**	**mean±s.d.**
1	Empty vector	100083	0	0.000	-
	Replicate 1	100152	370	0.369	0.347±0.193
	Replicate 2	100036	512	0.512	
	Replicate 3	100147	633	0.632	
	Replicate 4	100302	219	0.218	
	Replicate 5	99930	150	0.150	
	Replicate 6	100055	200	0.200	
2	Empty vector	100000	0	0.000	-
	Replicate 1	47180	1139	2.414	2.083±0.248
	Replicate 2	52604	1178	2.239	
	Replicate 3	91753	1632	1.779	
	Replicate 4	100000	2114	2.114	
	Replicate 5	100000	2146	2.146	
	Replicate 6	100000	1807	1.807	

Development • Supplementary information

3	Empty vector	100000	0	0.000	-
	Replicate 1	100000	814	0.814	0.859±0.227
	Replicate 2	100000	1332	1.332	
	Replicate 3	100000	673	0.673	
	Replicate 4	100000	820	0.820	
	vReplicate 5	100000	950	0.950	
	Replicate 6	100000	827	0.827	
	Replicate 7	100000	669	0.669	
	Replicate 8	100000	1051	1.051	
	Replicate 9	100000	596	0.596	
4	Empty vector	100003	6	0.006	-
	Replicate 1	100000	1250	1.250	1.047±0.140
	Replicate 2	100000	1091	1.091	
	Replicate 3	100000	1103	1.103	
	Replicate 4	100000	1049	1.049	
	Replicate 5	100000	849	0.849	
	Replicate 6	100000	938	0.938	

Development • Supplementary information

5	Empty vector	100229	9	0.009	-
	Replicate 1	100206	1048	1.046	1.204±0.128
	Replicate 2	100062	1352	1.351	
	Replicate 3	100070	1160	1.159	
	Replicate 4	100075	1368	1.367	
	Replicate 5	100067	1123	1.122	
	Replicate 6	100055	1182	1.181	
6	Empty vector	100000	0	0.000	-
	Replicate 1	100000	944	0.944	1.399±0.621
	Replicate 2	100000	472	0.472	
	Replicate 3	100000	1681	1.681	
	Replicate 4	100000	1802	1.802	
	Replicate 5	100000	2182	2.182	
	Replicate 6	100000	1315	1.315	
7a	Empty vector	12243	0	0.000	-
	Replicate 1	86084	1134	1.317	1.159±0.326
	Replicate 2	100000	559	0.559	
	Replicate 3	100000	1469	1.469	
	Replicate 4	100000	1376	1.376	
	Replicate 5	100000	1129	1.129	
	Replicate 6	100000	1101	1.101	
7b	Empty vector	12243	0	0.000	-
	Replicate 1	100000	980	0.980	1.094±0.148
	Replicate 2	100000	1151	1.151	
	Replicate 3	100000	1284	1.284	
	Replicate 4	100000	1118	1.118	
	Replicate 5	100000	1163	1.163	
	Replicate 6	100000	865	0.865	

Development • Supplementary information

Table S2. Flow Cytometry analysis of *Capsaspora* transfected cells during 10 days. Cells transfected with pONSY-Venus expression vector analysed every 24 hours during 10 days after transfection. Results from 3 independent experiments are shown. Transfection efficiency was calculated as the ratio of total number of positive cells (P+) from total number of cells (P2). Ratio of positive cells was calculated as the percentage of positive cells in a particular day relative to the percentage of positive cells at day 1 and represented as mean±s.d per day. Table associated to Fig. S4.

Experiment		Number of cells		Transfection efficiency		
Days post-transfection	Sample	Total (P2)	Positive (P+)	(P+/P2)%	Ratio %	mean±s.d.
1	Empty vector	21427	0	0.000	-	-
	Replicate 1	295873	313	0.106	100.000	100.000±0.000
	Replicate 2	99871	338	0.338	100.000	
	Replicate 3	99968	79	0.079	100.000	
2	Empty vector	99944	0	0.000	-	-
	Replicate 1	99962	32	0.032	30.260	38.898±8.303
	Replicate 2	99955	134	0.134	39.612	
	Replicate 3	100000	37	0.037	46.820	
3	Empty vector	99962	1	0.001	-	-
	Replicate 1	100000	22	0.022	20.796	14.260±7.044
	Replicate 2	99953	23	0.023	6.799	
	Replicate 3	100000	12	0.012	15.185	
4	Empty vector	100000	0	0.000	-	-
	Replicate 1	100000	8	0.008	7.562	6.731±1.466
	Replicate 2	99705	17	0.017	5.038	
	Replicate 3	100000	6	0.006	7.593	
5	Empty vector	100000	0	0.000	-	-
	Replicate 1	100000	8	0.008	7.562	7.823±4.706
	Replicate 2	99942	11	0.011	3.252	
	Replicate 3	100000	10	0.010	12.654	

6	Empty vector	100000	0	0.000	-	-
	Replicate 1	100000	5	0.005	4.726	3.854±1.809
	Replicate 2	99946	6	0.006	1.744	
	Replicate 3	100000	4	0.004	5.062	
7	Empty vector	100000	0	0.000	-	-
	Replicate 1	100000	11	0.011	10.398	6.757±3.446
	Replicate 2	99973	12	0.012	3.547	
	Replicate 3	100000	5	0.005	6.327	
8	Empty vector	100000	0	0.000	-	-
	Replicate 1	100000	9	0.009	8.508	5.859±3.822
	Replicate 2	99947	5	0.005	1.478	
	Replicate 3	100000	6	0.006	7.593	
9	Empty vector	235982	0	0.000	-	-
	Replicate 1	100000	12	0.012	11.343	4.723±5.841
	Replicate 2	99983	1	0.001	0.296	
	Replicate 3	100000	2	0.002	2.531	
10	Empty vector	186504	1	0.001	-	-
	Replicate 1	100000	6	0.006	5.672	3.031±2.430
	Replicate 2	99709	3	0.003	0.889	
	Replicate 3	100000	2	0.002	2.531	

Table S3. Flow Cytometry analysis of *Capsaspora* cells co-transfected with pONSY-Venus and pONSY-mCherry. Results from 7 independent experiments with 6 replicates each (n=42) are shown. Transfection efficiency is calculated by total number of positive cells (Q2+Q3+Q4) from total number of cells (P2) and represented as mean±s.d per experiment. Relative percentages of Double, Venus and mCherry expression were calculated as number of double positive cells (Q2) or number of Venus positive cells (Q4) or number of mCherry positive cells (Q3) from total number of positive cells (Q2+Q3+Q4), respectively, and represented as mean±s.d per experiment. Table associated to Fig. 3F and G and Fig. S5.

Experiment		Number of cells						Transfection efficiency		Relative % over total number of positive cells		
Number	Sample	Total (P2)	Negative (Q1)	mCherry (Q2)	Double (Q3)	Venus (Q4)	Total positive (Q2+Q3+Q4)	% Total positive	% Double (Q3/P2)%	% Double	% Venus	% mCherry
1	Empty vector	100000	99996	1	2	1	4	0.004				
	Control Venus	100000	98177	1	2	1820	1823	1.823				
	Control mCherry	100000	99689	310	1	0	311	0.311				
	Replicate 1	520488	516878	706	2346	558	3610	0.694	0.451	64.986	15.457	19.557
	Replicate 2	358753	355200	731	2354	468	3553	0.990	0.656	66.254	13.172	20.574
	Replicate 3	322368	319598	501	1818	451	2770	0.859	0.564	65.632	16.282	18.087
	Replicate 4	408411	404855	620	2339	597	3556	0.871	0.573	65.776	16.789	17.435
	Replicate 5	426129	422602	592	2357	578	3527	0.828	0.553	66.827	16.388	16.785
	Replicate 6	380788	377503	501	2305	479	3285	0.863	0.605	70.167	14.581	15.251
	Mean±s.d.							0.851±0.095	0.567±0.068	66.607±1.850	15.445±1.364	17.948±1.918
2	Empty vector	100000	99988	2	5	5	12	0.012				
	Control Venus	100000	98648	7	2	1343	1352	1.352				
	Control mCherry	100000	98879	1116	2	3	1121	1.121				
	Replicate 1	209708	206792	191	2247	478	2916	1.391	1.071	77.058	16.392	6.550
	Replicate 2	249962	247044	230	2217	471	2918	1.167	0.887	75.977	16.141	7.882
	Replicate 3	231155	228463	180	2154	358	2692	1.165	0.932	80.015	13.299	6.686
	Replicate 4	342982	340145	222	2220	395	2837	0.827	0.647	78.252	13.923	7.825
	Replicate 5	231457	228666	222	2203	366	2791	1.206	0.952	78.932	13.114	7.954
	Replicate 6	348075	345308	233	2167	367	2767	0.795	0.623	78.316	13.263	8.421
	Mean±s.d.							1.092±0.233	0.852±0.179	78.091±1.416	14.355±1.508	7.553±0.755
3	Empty vector	100677	100662	4	9	2	15	0.015				
	Control Venus	100712	99079	5	8	1620	1633	1.621				
	Control mCherry	100673	99677	990	5	1	996	0.989				
	Replicate 1	374093	371723	186	1881	303	2370	0.634	0.503	79.367	12.785	7.848
	Replicate 2	380307	377928	145	1940	294	2379	0.626	0.510	81.547	12.358	6.095
	Replicate 3	292266	289888	132	1972	274	2378	0.814	0.675	82.927	11.522	5.551
	Replicate 4	218046	215653	112	1910	371	2393	1.097	0.876	79.816	15.504	4.680
	Replicate 5	279028	276622	138	1937	331	2406	0.862	0.694	80.507	13.757	5.736
	Replicate 6	179012	176633	140	1892	347	2379	1.329	1.057	79.529	14.586	5.885
	Mean±s.d.							0.894±0.275	0.719±0.215	80.616±1.386	13.419±1.480	5.966±1.043

4	Empty vector	100000	100000	0	0	0	0	0				
	Control Venus	100000	99020	5	3	972	980	0.980				
	Control mCherry	100000	99794	205	1	0	206	0.206				
	Replicate 1	459409	456258	383	2359	409	3151	0.686	0.513	74.865	12.980	12.155
	Replicate 2	412692	409512	375	2388	417	3180	0.771	0.579	75.094	13.113	11.792
	Replicate 3	837030	833626	480	2436	488	3404	0.407	0.291	71.563	14.336	14.101
	Replicate 4	402481	399267	481	2387	346	3214	0.799	0.593	74.269	10.765	14.966
	Replicate 5	332644	329608	357	2352	327	3036	0.913	0.707	77.470	10.771	11.759
	Replicate 6	582324	579008	502	2398	416	3316	0.569	0.412	72.316	12.545	15.139
	Mean±s.d.							0.691±0.180	0.516±0.147	74.263±2.118	12.418±1.410	13.319±1.597
5	Empty vector	100000	99986	8	5	1	14	0.014				
	Control Venus	100000	99573	4	2	421	427	0.427				
	Control mCherry	100000	99983	16	1	0	17	0.017				
	Replicate 1	464014	461024	294	2300	396	2990	0.644	0.496	76.923	13.244	9.833
	Replicate 2	425876	423064	259	2300	253	2812	0.660	0.540	81.792	8.997	9.211
	Replicate 3	616927	613484	411	2300	732	3443	0.558	0.373	66.802	21.261	11.937
	Replicate 4	598473	595286	342	2300	545	3187	0.533	0.384	72.168	17.101	10.731
	Replicate 5	491626	488340	355	2300	631	3286	0.668	0.468	69.994	19.203	10.803
	Replicate 6	801753	798352	434	2300	667	3401	0.424	0.287	67.627	19.612	12.761
	Mean±s.d.							0.581±0.095	0.425±0.093	72.551±5.805	16.57±4.623	10.879±1.309
6	Empty vector	100262	100248	9	4	1	14	0.014				
	Control Venus	100194	99427	3	0	764	767	0.766				
	Control mCherry	100000	99676	324	0	0	324	0.324				
	Replicate 1	1172129	1168353	697	2517	562	3776	0.322	0.215	66.658	14.883	18.459
	Replicate 2	780326	776622	645	2520	539	3704	0.475	0.323	68.035	14.552	17.414
	Replicate 3	1036442	1032768	645	2513	516	3674	0.354	0.242	68.400	14.045	17.556
	Replicate 4	725427	721735	692	2511	489	3692	0.509	0.346	68.012	13.245	18.743
	Replicate 5	757172	753425	709	2531	507	3747	0.495	0.334	67.547	13.531	18.922
	Replicate 6	697333	693820	643	2460	410	3513	0.504	0.353	70.026	11.671	18.303
	Mean±s.d.							0.443±0.083	0.302±0.059	68.113±1.113	13.654±1.148	18.233±0.620
7	Empty vector	12243	12230	7	5	1	13	0.106				
	Control Venus	100000	98740	15	9	1236	1260	1.260				
	Control mCherry	100000	98809	1185	4	2	1191	1.191				
	Replicate 1	161940	160433	322	981	204	1507	0.931	0.606	65.096	13.537	21.367
	Replicate 2	155693	154319	172	1000	202	1374	0.883	0.642	72.780	14.702	12.518
	Replicate 3	123025	121624	186	1000	215	1401	1.139	0.813	71.378	15.346	13.276
	Replicate 4	163206	162117	174	783	132	1089	0.667	0.480	71.901	12.121	15.978
	Replicate 5	258248	256791	233	1000	224	1457	0.564	0.387	68.634	15.374	15.992
	Replicate 6	122506	121096	202	1000	208	1410	1.151	0.816	70.922	14.752	14.326
	Mean±s.d.							0.889±0.240	0.624±0.173	70.119±2.826	14.305±1.260	15.576±3.165

Development • Supplementary information

Table S4. List of primers used to build *Capsaspora* expression vectors with reporter genes. Restriction enzymes sites are underlined. CoNMM sequence plus 7 extra aminoacids is highlighted in red.

Region/Gene	*Capsaspora* gene ID	Primer name	Sequence 5'-3'
CoEF1α promoter	CAOG_07807	1	CTGGTACCAAATGCACAGTTAGCAACGACC
		2	GATATCACTAGTCCCGGGATCCTGTGAAGGTTGTTCTG
		3	AAATGCACAGTTAGCAACGACC
CoEF1α terminator	CAOG_07807	4	GAGCTGTACAAGTAAATTTTGTGTTTGCCAAG
		5	CATTGCTAGTGCTGTTCTCACC
		6	GACCGCGGTGAGAACAGCACTAGCAATG
		7	CCCGGGACTAGTGATATCTGAATTTTGTGTTTGCCAAGACAC
		8	CGCCAGTGTGATGGATTGAAAGCTTCCGCGGTGA
mCherry/Venus	-	9	CCCGGGACTAGTGATATCATGGTGAGCAAGGGCG
		10	CTTGGCAAACACAAAATTTACTTGTACAGCTC
CoSrc2 NMM	CAOG_06360	11	TATACCCGGG**ATGGGCTGCTCCAACTCTAAACCGCACGACCCGTCCGATTTCAAGGTTTCCCCTTCTGGCGTTGCGTCCAACAGC**ATGGTGAGCAAGGGCGAGGAG
		12	TTACTTGTACAGCTCGTCCATG
CoH2B	CAOG_01818	13	TACCCGGGATGCCGCCGAAGGTC
		14	TAACTAGTCTTGGCGCCGGAGGT
Lifeact	-	15	CCCGGGACCATGGGTGTGGCAGACCTGATTAAGAAGTTCGAGAGCATT
		16	TCTAGATGGTGGGTCACCCTCCTCCTTGCTAATGCTCTCGAACTTCTT

Development • Supplementary information
